# Elite Suppressor–Derived HIV-1 Envelope Glycoproteins Exhibit Reduced Entry Efficiency and Kinetics

**DOI:** 10.1371/journal.ppat.1000377

**Published:** 2009-04-10

**Authors:** Kara G. Lassen, Michael A. Lobritz, Justin R. Bailey, Samantha Johnston, Sandra Nguyen, Benhur Lee, Tom Chou, Robert F. Siliciano, Martin Markowitz, Eric J. Arts

**Affiliations:** 1 Department of Molecular Biology and Microbiology, Department of Medicine, Case Western Reserve University, Cleveland, Ohio, United States of America; 2 Division of Infectious Diseases, Department of Medicine, Case Western Reserve University, Cleveland, Ohio, United States of America; 3 Department of Medicine, Johns Hopkins University School of Medicine, Baltimore, Maryland, United States of America; 4 Department of Microbiology, Immunology, and Molecular Genetics, University of California Los Angeles, Los Angeles, California, United States of America; 5 Departments of Biomathematics and Mathematics, University of California Los Angeles, Los Angeles, California, United States of America; 6 Howard Hughes Medical Institute, Johns Hopkins University School of Medicine, Baltimore, Maryland, United States of America; 7 Aaron Diamond AIDS Research Center, Rockefeller University, New York, New York, United States of America; University of Zurich, Switzerland

## Abstract

Elite suppressors (ES) are a rare subset of HIV-1–infected individuals who are able to maintain HIV-1 viral loads below the limit of detection by ultra-sensitive clinical assays in the absence of antiretroviral therapy. Mechanism(s) responsible for this elite control are poorly understood but likely involve both host and viral factors. This study assesses ES plasma-derived envelope glycoprotein (*env*) fitness as a function of entry efficiency as a possible contributor to viral suppression. Fitness of virus entry was first evaluated using a novel inducible cell line with controlled surface expression levels of CD4 (receptor) and CCR5 (co-receptor). In the context of physiologic CCR5 and CD4 surface densities, ES *envs* exhibited significantly decreased entry efficiency relative to chronically infected viremic progressors. ES *envs* also demonstrated slow entry kinetics indicating the presence of virus with reduced entry fitness. Overall, ES *env* clones were less efficient at mediating entry than chronic progressor *envs*. Interestingly, acute infection *envs* exhibited an intermediate phenotypic pattern not distinctly different from ES or chronic progressor *envs*. These results imply that lower *env* fitness may be established early and may directly contribute to viral suppression in ES individuals.

## Author Summary

The majority of HIV-1–infected individuals experience high plasma viral loads and CD4^+^ T cells loss in the absence of antiretroviral therapy. However, a very rare and important subset of individuals termed elite suppressors is able to maintain HIV-1 plasma viral loads below the limit of viral detection in the absence of treatment. The reasons behind this ability to control the virus are poorly understood, but they likely involve both an effective host immune response against HIV-1 and factors related to the virus itself. Here, we analyze the function of the HIV-1 coat protein or envelope glycoprotein from a group of elite suppressors. HIV-1 envelope mediates entry into the host cell via interaction with the cellular receptors CD4 and CCR5. Envelopes from elite controllers interacted with these receptors inefficiently compared to those from individuals with detectable viral loads. These inefficient interactions by elite suppressor envelopes led to slow rates of entry into host cells. Envelopes from acutely infected individuals were not significantly different from elite suppressors or chronically infected individuals. These findings suggest that the decreased envelope efficiency may contribute to viral control in elite suppressors.

## Introduction

A minor subset of HIV-1–infected individuals maintains stable CD4^+^ T cell counts in the absence of antiretroviral therapy. A small proportion of these long-term nonprogressors (LTNPs), termed elite suppressors (ES), control plasma viral loads to <50 copies/ml [1]. Mechanism(s) responsible for this elite control are poorly understood but likely involve host and viral factors. Studies have explored the contributions of the innate and adaptive immune responses, host genetic polymorphisms, and viral dynamics (reviewed in [2]). For example, the major histocompatibility complex class (MHC) I group B alleles HLA-B27, -B51, and –B57 have been strongly associated with slower rates of HIV-1-associated disease progression [3]–[6]. Although these HLA-B alleles are overrepresented in ES and LTNPs, they are only expressed in a subset of these individuals indicating that the presence of these alleles is not necessary to suppress viremia and that other factors are likely involved [4],[7].

Although much previous work on ES has focused on host factors, less is known about viral fitness in these individuals. The impact of viral attenuation on disease progression was first described in a cohort of LTNPs infected by a common donor with virus containing a deletion in the *nef* gene [8],[9]. Investigation of other LTNP cohorts has shown both the presence [10],[11] and absence [12],[13] of defective *nef* genes. In other cohorts, the presence of viruses with reduced replication capacity has been associated with slower disease progression [14]–[19]. This viral attenuation could be the result of divergent evolution as a result of direct selective pressure by the host immune response [16]–[19]. However, recent work has shown that replication-competent viruses can be recovered from ES individuals indicating that ES harbor functional virus [20]. Furthermore, large scale sequencing of ES viruses yielded no identifiable common genetic defects [21]. Investigating the relative fitness of viral quasispecies in ES will help determine whether viral fitness is influencing disease outcome in these individuals.

Low HIV-1 genetic diversity in ES may be indicative of the presence of lower fitness variants [22]. Sequence analysis of functional envelope glycoprotein ES clones showed significantly decreased *env* diversity compared to individuals with chronic viremia suggesting that viruses in these patients experience minimal viral replication and diversification [23]. Lack of *env* diversification suggests that ES *envs* may be closely related in genotype and phenotype to the founder virus establishing infection.

In this study we have performed rigorous phenotypic analysis on subtype B *env* clones from ES plasma virus to determine whether *env* fitness may be contributing to viral suppression in ES. A novel cell line was utilized to show that ES *env* clones exhibit low CD4 receptor and CCR5 co-receptor usage and slow fusion kinetics compared to chronic infection *envs*. Analysis of control viruses indicated that these characteristics directly correlated to reduced replication capacity *in vitro*. Acute infections *envs* were intermediate in their entry efficiency and not significantly different from either chronic or ES *envs*. This study provides the first direct evidence that decreased *env* function is a property of ES and that this may contribute to viral suppression.

## Materials and Methods

### Ethics statement

This study was conducted according to the principles expressed in the Declaration of Helsinki. The study was approved by the Institutional Review Board of Johns Hopkins School of Medicine and Rockefeller University hospitals. All patients provided written informed consent for the collection of samples and subsequent analysis.

### Patient profiles

The elite suppressor and chronic progressor patients have been previously described [23] (Table 1). Patients identified with acute/early HIV-1 infection have been previously described [24]. The estimated duration of infection was calculated 2 weeks prior to the onset of acute retroviral illness unless the patient could identify a precise high risk event. Table 1 contains relevant enrollment data for all acute/early infection patients. Elite suppressors were defined as individuals who maintained viral load to below 50 copies of RNA/ml plasma in the absence of retroviral therapy yet were Western blot positive for infection. Informed consent was obtained prior to phlebotomy. The protocol for ES/CP or acute/early infection was approved by an institutional review board of the Johns Hopkins University School of Medicine and the Aaron Diamond AIDS Research Center, respectively.

**Table 1:** Patient characteristics of elite suppressors, chronic progressors, and acutely infected individuals.

Patient	Year of diagnosis	Sampling date	Duration of infection^a^	Plasma viral load (RNA copies/ml)	CD4 count (cells/µl)
**Elite Suppressors**					
ES2	1986	5/04	18 years	<50	383
ES3	1991	3/04	13 years	<50	677
ES4	1996	8/04	8 years	<50	837
ES7	1994	1/05	11 years	<50	1,125
ES8	2003	6/04, 9/04	1 year	<50	458
ES9	1999	3/04, 8/04	5 years	<50	800
ES10	2002	3/04	2 years	<50	900
**Chronic Progressors**					
C61	1999	9/04	5 years	19,100	1,261
C62	1998	9/04	6 years	33,300	481
C93	2001	3/05	4 years	47,270	402
C94	1999	2/05	6 years	22,898	351
C96	2001	3/05	4 years	12,500	400
C98	2004	3/05	1 year	17,838	426
C109	2002	5/05	3 years	61,000	222
**Acute Infection**					
502	–	–	19 days	31,622,777	306
503	–	–	17 days	588,844	531
504	–	–	32 days	2,691,535	152
506	–	–	43 days	467,735	341
508	–	–	26 days	389,045	266
510	–	–	16 days	1,584,893	581
512	–	–	25 days	3,388,442	745
514	–	–	26 days	676,083	291
516	–	–	51 days	295,121	371
517	–	–	38 days	87,096	588
518	–	–	15 days	21,379,621	226
519	–	–	22 days	1,819,701	322
520	–	–	40 days	257,040	512
522	–	–	21 days	3,235,937	583
523	–	–	28 days	173,780	273
526	–	–	26 days	100,000	205
527	–	–	21 days	134,896	538
528	–	–	16 days	12,022,644	438
529	–	–	19 days	28,840,315	537
530	–	–	30 days	6,456,542	469

a Time of infection expressed in approximate years for ES and CP patients from diagnosis to sampling. Time of infection for acute infection samples estimated by 14 days after the onset of acute seroconversion symptoms or identification of high risk transmission event, and sampling was conducted <4 weeks post-presentation and initiation of HAART.

### Generation of pseudotype virus and infection of Affinofile cells

Envelope expression vectors were generated as previously described [23]. Envelope pseudotypes were generated by cotransfection of 293T cells with the 1 µg of the luciferase-encoding pseudotyping vector pNLLuc.AM and 1 µg of envelope expression vector. Cells were washed after 24 h, and pseudoviruses were collected after a subsequent 48 h. Relative particle numbers were determined by limiting dilution reverse transcriptase assay. Viruses were characterized as exclusively CCR5-utilizing by comparison of infectivity of U87-CD4/CCR5 and U87-CD4/CXCR4 cells, as previously described [25]. Affinofile cells were generated by selection of 4 vector stable cells (Johnston *et al.*, submitted). CCR5 expression is controlled by a two vector ecdysone-inducible promoter. pVgRXR encodes the VgEcR fusion protein under control of the CMV promoter, and the RXR open reading frame under control of the RSV 5′ long terminal repeat. pIND-CCR5 encodes CCR5 under control of the minimal heat shock promoter with inducible control provided by five repeats of the glucocorticoid receptor DNA binding domains (5×E/GRE). Addition of the ecdysone derivative ponasterone A (the inducer) results in recruitment of a transcriptional coactivator to the 5×E/GRE element and activation of transcription of the CCR5 ORF. CD4 expression is inducibly regulated by the TREx expression system (Invitrogen). Cells contain pcDNA5-TO-CD4 and transcription of the CD4 ORF is controlled by the addition of the tetracycline analog minocycline. Single cell clones were isolated to generate cell populations with consistent levels of induction upon stimulation of CD4 and CCR5 expression.

Affinofile cells were plated at a density of 10,000 cells per well in a 96-well plate and allowed to adhere for 48 hours. Cells were induced in a matrix pattern to express CD4 and CCR5. Minocycline was added to cells in 2-fold dilutions over 6 separate dilutions (5 ng/ml–0 ng/ml) to induce CD4 expression. Ponasterone A was added in 2-fold dilutions over 6 separate dilutions from a final concentration of 4 µM to 0 µM to induce CCR5 expression. This matrix results in 36 unique CD4 and CCR5 induction surface concentrations. Each drug concentration was induced in triplicate. Cells were induced for 24 hours prior to infection. Cells were then exposed to pseudovirus for 48 h, washed with PBS, and lysed with Glo lysis buffer (Promega, Inc.). Maximal infection was considered luciferase activity generated by infection at the highest CD4 and highest CCR5 concentration. To control for effects caused directly by minocycline and/or ponasterone A on viral infectivity, U87-CD4/CCR5 cells were treated with a similar matrix of both drugs. CCR5 and CD4 expression levels were unchanged by flow cytometry, and no changes in infectivity of Yu-2 and SF162 envelope pseudoviruses were noted, thus variation in infectivity was assumed to be due to variations in receptor expression levels (Johnston *et al*, submitted).

### Kinetic fusion and reverse transcription assays

For the kinetic fusion assay, HIV-1 pseudoviruses bearing either ES or chronic envelopes were spinonculated onto U87-CD4/CCR5 cells. 2.5×10^6^ cells were spin-infected with pseudovirus-containing supernatant for 90 min at 1,200×g at 4°C. The cells were washed twice with cold phosphate buffered saline (PBS) to remove unbound virions. Cells were resuspended in cold medium and split into 96-well plates (50 µl/well). Virus-cell mixes were synchronized for entry by addition of 130 µl of 37°C medium, and then ENF at 10 µM was added to each well in 20 µl of medium at fixed-time intervals after addition of warm medium, which is defined as tim = 0 for synchronization of viral replication. Cells were incubated for 48 h and then treated with lysis buffer and luciferase activity was determined. For the kinetic fusion assay 6 hours was used as 100% or maximal luciferase activity. For the reverse transcription assay, Affinofile cells were induced with 5 ng/ml minocycline 24 hours prior to infection. ES or chronic pseudoviruses were synchronously added to cells. Efavirenz (EFV) was added at a concentration of 1 µM to each well at fixed time intervals after the addition of virus. For reverse transcription assay, 12 hours was used as 100% or maximal luciferase activity.

### Entry inhibitor susceptibility

Affinofile cells were induced 24 hours prior to infection with 5 ng/ml minocycline. Cells were incubated with serial 10-fold dilutions of either chemokine (CCL5 [50 nM to 0.1 nM]) or drug (ENF (T-20) [1 µM to 0.1 nM], TAK-779 [1 µM to 0.1 nM]) for 1 h prior to the addition of virus. Cells were incubated for 48 h, washed with PBS, lysed, and luciferase activity determined. Plots of luciferase activity versus drug concentration were used to determine IC_50_ values for each pseudovirus. Luciferase activity without drug was used as maximal or 100% infection value.

### Flow cytometry

Peripheral blood mononuclear cells (PBMCs) were isolated from heparin-treated venous whole blood from HIV–seronegative donors by Ficoll-paque density gradient centrifugation (GE Healthcare, Piscataway, NJ). Isolated PBMCs were washed twice in wash buffer [phosphate-buffered saline (PBS) supplemented with 2% fetal bovine serum (FBS), 0.1% glucose, 12 mM HEPES, and penicillin (100 U/m) and streptomycin (100 µg/ml)] and activated in RPMI 1640 (Mediatech, Inc., Manassas, VA) supplemented with 10% FBS, penicillin/streptomycin, and 2 µg/ml phytohemmaglutinin (PHA, Sigma-Aldrich, St. Louis, MO) and 100 U/ml interleukin 2 (IL-2, Invitrogen, Carlsbad, CA) for 3 days at 37°C and 5% CO_2_. Total PBMCs were subsequently maintained in RPMI 1640 supplemented with 10% FBS, pen/strep, and 100 U/ml IL-2. For flow cytometry experiments, a total of 10° PBMCs were collected 4 days post-stimulation by centrifugation at 2500 rpm×10 minutes and washed once in FACS staining buffer (PBS with 2% FBS, 0.5% bovine serum albumin, and 0.02 sodium azide). The cells were incubated in either an anti-CD4 antibody [Fluorescein isothiocyanate(FITC)-conjugated anti-CD4, BD Biosciences Pharmingen, San Jose, CA] or FITC-conjugated IgG_1_ isotype control (BD Biosciences Pharmingen), or an anti-CCR5 antibody [Phycoerythrin(PE)-conjugated anti-CCR5 clone CTC5, R&D Systems, Minneapolis, MN] or PE-conjugated IgG_2B_ isotype control (R&D Systems). All antibodies were incubated at a final concentration of 12.8 µg/ml for 30 minutes at room temperature in FACS staining buffer. Stained PBMCs were washed again in FACS staining buffer and samples were analyzed on a FACSCalibur flow cytometer (Becton Dickinson, Franklin Lakes, NJ) with Cellquest software. For assessment of Affinofile cell receptor expression levels relative to PBMCs, 5.0×10^5^ cells were added to a 6-well plate and allowed to adhere for 48 hours in Dulbecco's Modified Eagle's Medium (DMEM, Mediatech, Inc.) supplemented with 10% FBS, pen/strep, and 50 µg/ml blasticidin (Sigma). Cells were stimulated with 20 ng/ml minocycline (Sigma) for 24 hours and subsequently recovered from plates with 3 mM EDTA in PBS. Cells were washed, stained, and analyzed by flow cytometry as reported above for PBMCs. Flow cytometry of PBMCs and Affinofile cells was performed in the same experiment. Results were analyzed by Flow-Jo software and receptor expression levels reported as events relative to mean fluorescence intensity.

### Pseudovirus incorporation of envelope glycoproteins

Envelope-pseudotyped viruses prepared with ES or CP envelopes by transfection of 293T cells were quantified by limiting dilution reverse transcriptase activity. Equivalent virion numbers were pelleted by centrifugation at 38,000×g for 2 hours at 4°C. Supernatant was removed and virion pellets were lysed in SDS lysis buffer [40 mM Tris-HCl (pH 6.8), 10% glycerol, 10% ß-mercaptoethanol, 1% SDS]. Virus lysates were separated on SDS-10% polyacrylamide gels and transferred to nitrocellulose. Membranes were blocked with gelatin and proteins were detected either with a mouse monoclonal anti-gp120 antibody that recognizes a conserved C2 region linear epitope (B13, courtesy of Dr. Bruce Chesebro, NIAID ) or HIV-Ig (courtesy AIDS Research and Reference Reagent Program). Primary antibodies were detected with horseradish peroxidase-conjugated goat-anti-mouse or goat-anti-human secondary antibodies, respectively (Pierce Biotechnology, Rockford, IL), revealed with the ECL Plus Western Detection kit (Pierce Biotechnology) and exposed to X-ray film.

### Statistical analysis

Data were analyzed by the UCLA Statistical/Biomathematical Consulting Clinic using repeated measures ANOVA, simultaneously taking into account effects due to disease group (ES vs CP), CD4 level, and CCR5 level. Within each group, the intrapatient variability in relative infection was small and did not differ significantly between the ES and CP groups. Therefore, results are reported with interpatient variance. For evaluation of surface plots between groups ES and CP, *P* values are given using the average of clones for a given patient as a single value or using each individual clone as a single value. For groups of 3 or more (ES, CP, and acute) we evaluated independent means by One-way ANOVA using the Kruskal-Wallis test and Dunns post-test for data that did not pass a normalcy test. For drug sensitivity and kinetic analysis statistics were performed using each individual clone as a single value. We considered a *P* value of <0.05 as statistically significant.

## Results

### Measuring HIV-1 entry efficiency and fitness by varying CD4 and CCR5 cell surface concentrations

The ability of HIV-1 to infect a cell is largely influenced by surface expression of CD4 and CCR5 [26]–[32]. This study evaluates a previously described cohort of 38 independent full-length plasma *env* clones derived from 7 ES individuals [23]. The *env* clones from this cohort expressed similar levels of protein by Western blot (Figure S1) and readily infected the indicator cell line TZM-bl demonstrating their functionality [23]. As described below, observed differences in entry efficiency could not be explained by any minor variations in Env levels on the virus.

**Figure 1:**
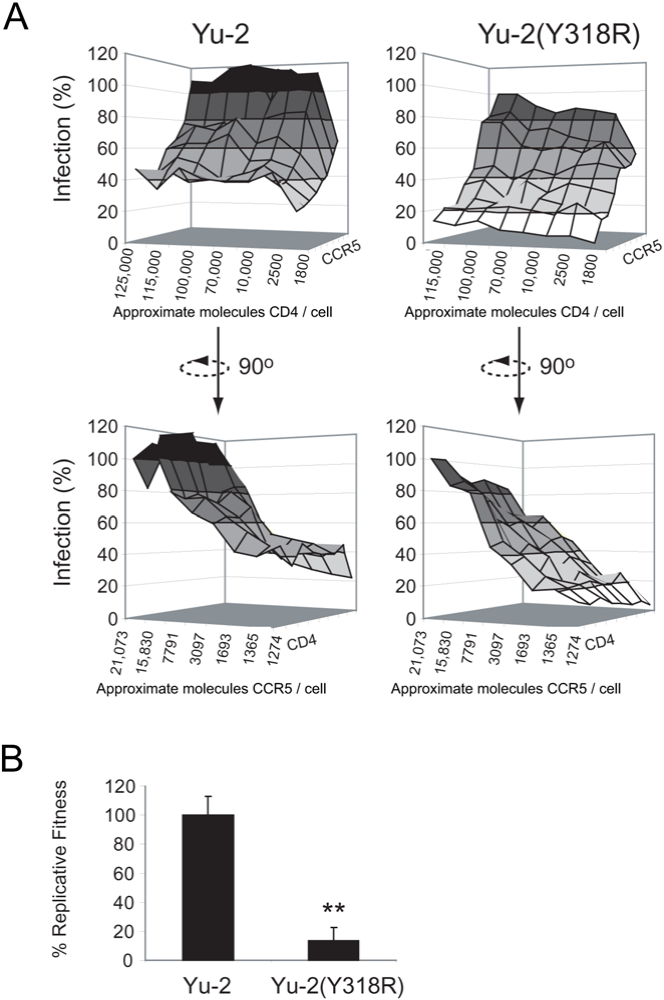
Affinofile cells allow for the analysis of both CD4 and CCR5 usage and are a surrogate marker of fitness. (A) Affinofile cells were infected with Luciferase-encoding envelope psudotyped viruses bearing the envelope of the neurotropic lab strain Yu-2 or a V3-crown mutant [Yu-2(Y318R)] with reduced CCR5 usage. Surface plots were constructed using infectivity data derived from luciferase activity. Percent infection (y-axis) was derived from the luciferase activity at each receptor expression combination divided by the luciferase activity at maximal CD4/CCR5 expression levels. The x-axis depicts approximate CD4 expression level described as approximate molecules CD4/cell as assessed by quantitative flow cytometry (top 2 panels), and the z-axis depicts approximate CCR5 expression levels (bottow 2 panels). (B) Relative replicative fitness of full length HIV-1 viruses in multiple replication cycle in primary PHA/IL-2–activated peripheral blood mononuclear cells. The replicative fitness of full length Yu-2 and Yu-2(Y318R) was determined by pairwise infection and quantification of relative outgrowth. The y-axis represents percent replicative fitness of Yu-2(Y318R) relative to Yu-2.

As with most cell lines, TZM-bls express CD4 at levels comparable to primary activated CD4^+^ T cells (approximately 65,000–100,000 molecules/cell), however CCR5 expression is significantly higher than on primary T lymphoctyes (approximately 500 to 7000 molecules/cell) [26], [33]–[37]. CCR5 expression also varies widely from patient to patient not only in absolute number of cells expressing CCR5, but also in CCR5 density/cell [26], [27], [33]–[35]. This study utilizes the Affinofile system, a novel cell line with independent dual-inducible surface expression of CD4 and CCR5 (Figure 1, Figure S1) (Johnston *et al.*, submitted). The ability to modulate receptor and co-receptor expression on the Affinofile cells provides a more physiologic measure of HIV-1 entry efficiency. Since the ability of HIV-1 to infect a cell is largely influenced by cell surface levels of CD4 and CCR5, it is important to consider expression levels when evaluating infectivity [26]–[32].

Receptor usage as measured by the Affinofile system was validated as a surrogate marker of entry fitness. Yu-2 and a V3 crown mutant of Yu-2 [Yu-2(Y318R)] known to affect CCR5 usage were evaluated for infectivity using Affinofile cells induced at each pairwise combination of [Minocycline] and [Ponasterone A] (42 unique combinations) (Figure S1). Three-dimensional surface plots were generated from luciferase activity expressed as a function of virus infectivity at each combination of CCR5 and CD4, which was confirmed by flow cytometry (Figure 1A). CD4 and CCR5 surface levels at each drug combination are given as an average level calculated from a pool of cells expressing a range of CD4 and CCR5 molecules (Figure S4). Reduced infectivity of the Yu-2(Y318R) variant over the wild type was observed over a range of CCR5 and CD4 (Figure 1A). This decreased ability of Yu-2(Y318R) to infect cells expressing low CCR5 is consistent with a 90% reduction in replicative fitness measured by competitive replication assays in peripheral blood mononuclear cells (PBMCs) (Figure 1B, p<0.01, unpaired student's t-test). The direct relationship between entry efficiency using the Affinofile system and replicative fitness in human PBMCs has been validated for multiple primary HIV-1 isolates. Generally, viruses of increased replicative fitness display increased infectivity of cells expressing low CCR5, CD4, or both CCR5 and CD4 in the Affinofile system (Johnston *et al*, submitted).

To assess relative infectivity of chronic and ES *env* clones, pseudotyped viruses carrying a non-LTR driven luciferase were generated for each clone. Pseudotyped viruses generated from 38 independent plasma virus *env* glycoprotein clones from 7 ES and 32 independent plasma virus clones from 7 chronic progressors (CP) were evaluated for infectivity at each pairwise drug combination described above and surface plots were generated (Figure S2 and Figure S3). The percent infection defines the infection at each surface CCR5/CD4 level relative to a 100% infection at the highest CD4 and CCR5 surface density. This method permits the direct comparison of CD4 and CCR5 usage by each *env* clone and provides a rapid and efficient way to measure viral *env* replicative fitness.

### ES are less efficient than CP *env* clones in utilizing both CCR5 and CD4 for entry

**Figure 2:**
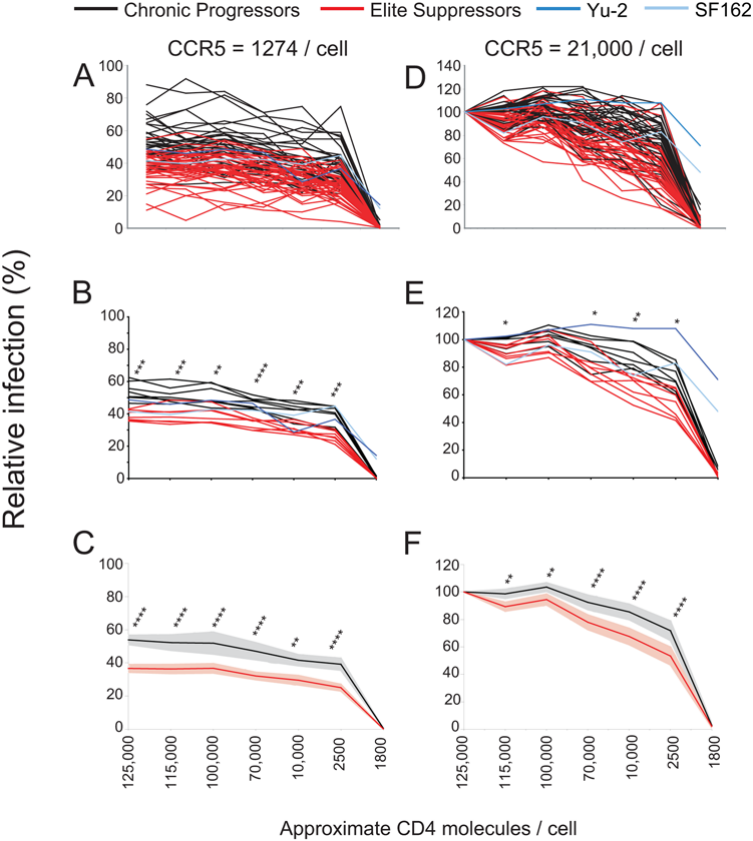
ES clones show reduced entry efficiency at fixed CCR5 surface concentrations. (A–C,D–F) Affinofile cells were induced to express approximately 1274 CCR5 molecules/cell (A–C) or approximately 21,073 CCR5 molecules/cell (D–F) and CD4 molecules ranging from approximately 1,800 to 125,000 molecules/cell prior to infection. Induced Affinofile cells were infected with equivalent amounts of pseudotyped viruses. Relative infection of all CP (black) and ES (red) clones are shown with SF162 and YU-2 included as positive controls (blue). Relative infection is expressed as a proportion of maximal infection at the highest CD4 (approximately 125,000 molecules/cell) and CCR5 (approximately 21,073 molecules/cell) level. (B,E) Infectivity of clones from individuals were averaged and plotted as a single line for chronic and ES individuals. P values are given for patient averages. (n = 7) (C,F) Average of all individual chronic or elite clones at minimal (C) and maximal (F) CCR5 concentrations with CD4 concentrations ranging from approximately 1,800 to 125,000 molecules/cell. Affinofile cells were induced as described above for (A,B). *P* values as calculated by unpaired student's *t* test are shown above each CD4 expression condition. *P* values are represented as follows: *P*<0.05 (*), *P*<0.01 (**), *P*<0.001 (***), *P*<0.0001 (****). 95% confidence intervals are shown for chronic (grey) and ES (pink).

**Figure 3:**
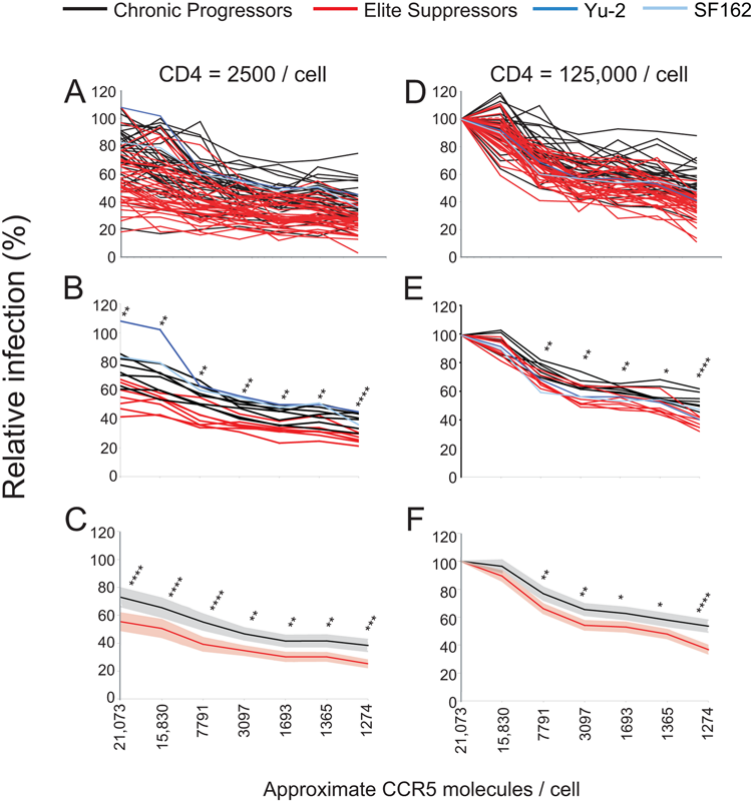
ES clones show reduced entry efficiency at fixed CD4 surface concentrations. (A–C,D–F) Affinofile cells were induced to express threshold (approximately 2,500) CD4 molecules/cell (A–C) or maximal (approximately 125,000) CD4 molecules/cell (D–F) and CCR5 molecules ranging from approximately 1274 to 21,073 molecules/cell prior to infection. Infection is performed as described in Figure 2. (B,E) Infectivity of clones from individuals were averaged and plotted as a single line for chronic and ES individuals. P values are given for patient averages. (n = 7) (C,F) Average of all individual chronic or elite *env* clones at threshold (C) and maximal (F) CD4 concentrations with CCR5 concentrations ranging from approximately 1274 to 21,073 molecules/cell. *P* values are represented as follows: *P*<.05 (*), *P*<.01 (**), *P*<.001 (***), *P*<.0001 (***). 95% confidence intervals are shown for chronic (grey) and ES (pink).

Relative infectivity of 38 independent ES *env* clones and 32 independent CP *env* clones was ascertained at multiple combinations of CCR5 and CD4 density (Figure 2A–2F and Figure 3A–3F). Values for each of the *env* clones tested are shown as well as for Yu-2 and SF162. Varying CD4 levels with constant CCR5 (Figure 2A–2F) or varying CCR5 levels with constant CD4 (Figure 3A–3F) consistently demonstrated that ES *env* clones supported lower levels of infection than the CP clones.

At the highest CD4 and lowest CCR5 expression level, ES clones averaged 36.7% while CP clones were reliably higher averaging 53.3% (Figure 2A–2C). Infectivity differences were significant for each CD4 concentration (*P* values ranged from 0.01 to <0.0001, repeated measures ANOVA) at a fixed high or low CCR5 level regardless if individual *env* clones were evaluated (Figure 2C and 2F) or if the *env* clones were averaged for a given individual and compared as patient averages (Figure 2B and 2E). Additionally, consistent with previous data, the neurotropic *envs* SF162 andYU-2 readily infected cells expressing sub-threshold levels of CD4 while the primary isolates could not (Figure 3A–3F) [38]. Taken together, these results reveal that ES clones inefficiently infect cells expressing low CCR5 in the presence of threshold or higher levels of CD4 compared to CP clones. Additionally, the discrepancy in infectivity between ES and chronic clones at fixed, high CCR5 levels indicates that ES clones also require higher levels of CD4 to achieve similar infection as chronic clones.

To further evaluate CCR5 usage independent of CD4 expression, infection was determined at minimal and maximal CD4 levels as CCR5 expression was varied. At each concentration of CCR5 below maximal examined, ES clones infected significantly less efficiently than chronic clones (Figure 3D–3F). Therefore, even in the presence of optimal CD4 concentrations, ES clones inefficiently utilize CCR5 for entry.

### ES *env* clones do not differ significantly from acute infection *env* clones in CD4 and CCR5 utilization

**Figure 4:**
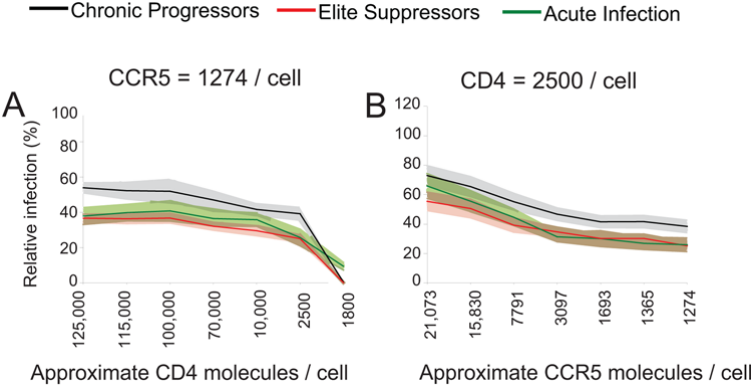
Acute *env* clones show no significant receptor or co-receptor utilization difference from chronic or elite envs. (A) Affinofile cells were induced to express minimal CCR5 (approximately 1274 molecules/cell) and CD4 molecules ranging from approximately 1,800 to 125,000 molecules/cell or (B) threshold (approximately 2,500) CD4 molecules/cell and CCR5 molecules ranging from approximately 1,274 to 21,073 molecules/cell prior to infection. Induced Affinofile cells were infected with equivalent amounts of pseudotyped viruses. Average of all acute (green), chronic (black), and elite (red) clones are shown. 95% confidence intervals are given for acute (light green), chronic (grey) and ES (pink).

Differences in receptor and co-receptor utilization of ES and chronic progressor *envs* were most significant when infection was performed in the context of (1) low CCR5 and varying CD4 levels or (2) low CD4 and varying CCR5 levels. Thus, these conditions were repeated to examine entry efficiency of 23 pseudotyped *env* plasma clones from 20 acutely infected individuals (Figure 4A and 4B). At low CCR5 expression, acute *envs* averaged an intermediate pattern of infectivity compared to ES and chronic *envs*, but these differences were not significant (Figure 4A). Similar results were obtained when infections were performed at minimal surface CD4 levels (Figure 4B). Consistent with previous reports using similar systems, these results indicate that acute *envs* show a broad pattern of infectivity which is not significantly different from chronic or ES *envs* [39].

### Susceptibility of *env* clones to CCL5, TAK-779, or ENF

**Figure 5:**
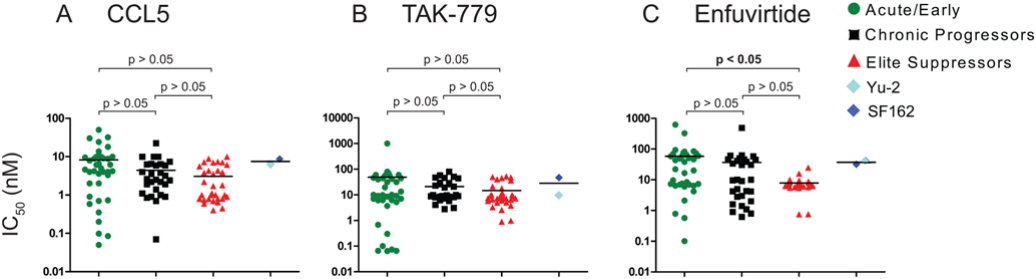
ES, CP, and acute clones show no differential susceptibility to CCL5, TAK-779, or ENF. (A–C) ES, acute, and chronic clones were used to infect Affinofile cells induced to express approximately 125,000 molecules CD4 and approximately 1,274 molecules CCR5/cell. (A) CCL5 was added in concentrations ranging from 0.1–50 nM, incubated for 1 h, and infected. Plots represent percent replication relative to no drug. 50 percent inhibitory concentrations of drug (IC_50_ values) were computed from the curves and plotted for acute, chronic, ES, and Yu-2 or SF162 envelopes. (B) TAK-779 was added to the induced Affinofile cells at concentrations ranging from 0.1 nM–1 µM. Plots were used to determine IC_50_ values for each envelope. (C) Enfuvirtide was added to cells at concentrations ranging from 0.1 nM–1 µM.

Previous studies suggest that major differences in entry efficiency may impact on susceptibility to various entry inhibitors [25], [40]–[42]. Thus ES, CP, and acute clones were tested for their susceptibility to the natural CCR5 ligand CCL5 (RANTES), the small molecule CCR5 antagonist TAK-779, and the fusion inhibitor enfuvirtide (ENF) in Affinofile cells induced to express CD4 and CCR5 to levels that closely mimic primary CD4^+^ T cells (approximately 125,000 molecules CD4/cell and 1274 molecules CCR5/cell) (Figure S4). Susceptibility to CCL5 did not differ significantly between chronic and ES *env* clones (Figure 5A). Interestingly, acute clone IC_50_ values ranged from 0.05 to 50 nM (1,000-fold) with an average of 8.25 nM. The range of IC_50_ values for acute clones was much larger relative to ES clones whose values ranged from 0.4 to 10 nM (25-fold), with an average of 3.05 nM. Although similar trends were observed with the small molecule CCR5 antagonist TAK-779, clones were not significantly different with average IC_50_ values of 48.8 nM for acute, 21.0 nM for chronic, and 14.7 nM for ES (Figure 5B). These results show that ES, CP, and acute *envs* have no remarkable differential susceptibility to CCL5 or TAK-779 however the broad range of IC_50_ values for acute *envs* highlights the variability in *env* phenotypes associated with acute infection.

Finally, susceptibility of clones to ENF was evaluated (Figure 5C). IC_50_ values for acute clones again showed a broad range from 0.10 to 623 nM (>5000 fold range) with an average of 58.13 nM. Again, ES (33-fold range) clones exhibited a significantly narrower range of IC_50_ values compared to acute clones. The average IC_50_ value for acute *envs* was significantly greater than for ES (*P*<0.05, ANOVA, Kruskal-Wallis test). Overall, ES clones showed trends towards increased susceptibility to entry inhibitors consistent with decreased entry efficiency. These susceptibility profiles suggest that ES clones have a high degree of phenotypic similarity indicated by the narrow range of susceptibility to entry inhibitors. Conversely, chronic and especially acute *envs* showed broad ranges of susceptibility indicative of their diverse entry phenotypes.

### Delayed fusion and reverse transcription kinetics exhibited by ES–derived *env* clones

Infection data of cells expressing sub-maximal concentrations of CD4 and CCR5 indicates that ES-derived *env* clones require higher levels of receptor and co-receptor for efficient entry. This requirement for higher receptor levels could suggest that these *envs* also exhibit differences in the rates of HIV-1 entry into host cells [42],[43].

**Figure 6:**
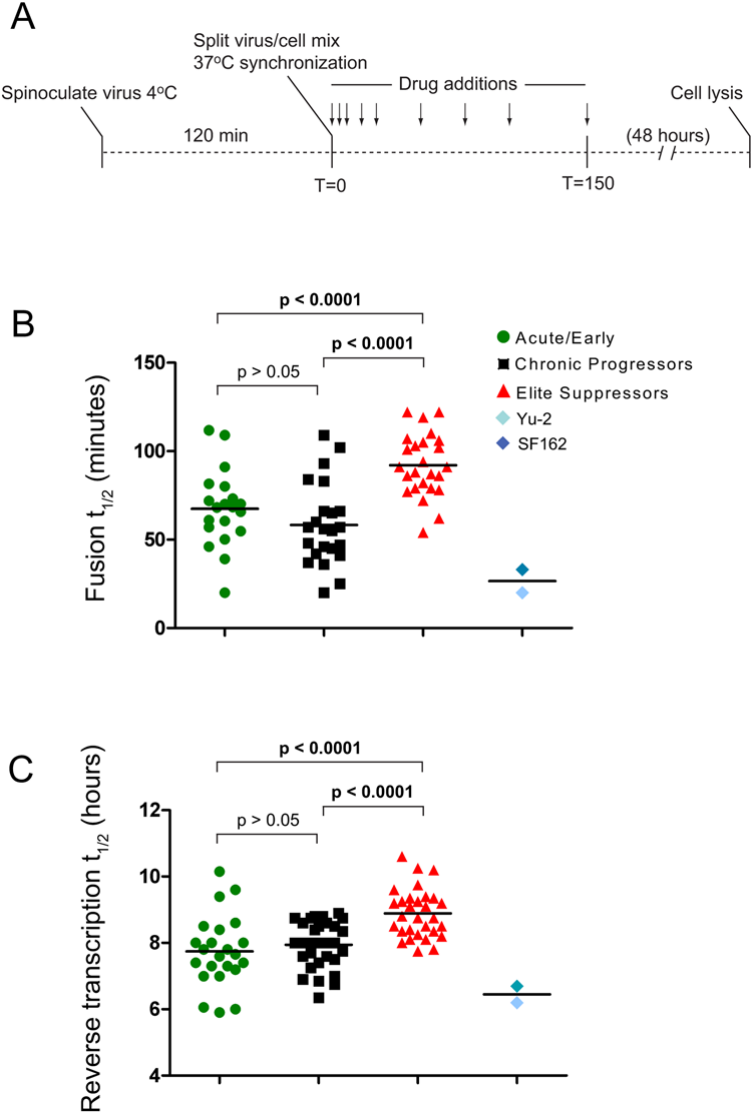
ES clones show slower fusion and reverse transcription kinetics compared to chronic and acute clones. (A) U87-CD4/CCR5 cells were spinoculated with equivalent amounts of pseudotyped virus at a temperature non-permissive for entry. Infections were synchronized by simultaneous addition of warm medium. ENF (10 µM) was added once to wells at given times post-infection. All infections are given as a percent of maximal infection. (B) Average T_1/2_ fusion values in minutes for acute (green), chronic (black), and ES (red). (C) Equivalent amounts of pseudotyped virus were simultaneously added to Affinofile cells induced to express approximately 125,000 molecules CD4 and 1274 molecules CCR5/cell. EFV (1 µM) was added at various times post-infection. Average T_1/2_ reverse transcription values in hours are shown. YU-2 and SF162 are included as controls (B,C).

To assess host cell entry kinetics, U87-CD4/CCR5 cells were first spinoculated with virus at a temperature non-permissive for viral fusion with the host cell. Enfuviritide (ENF) was added once to each well at a concentration of 10 µM at various times after the cells were shifted to temperatures permissive for viral fusion (Figure 6A). Viruses that have completed the final step in HIV-1 entry (six helix bundle formation) are ENF insensitive and will continue the viral replication cycle regardless of the addition of ENF. Thus, this assay permits determination of the entry kinetics of each *env* clone.

Fusion kinetics were measured for each ES, CP, and acute clone. ES clones fused with an average T_1/2_ of 92.1 minutes while acute clones averaged a T_1/2_ of 67.5 minutes and chronic clones a T_1/2_ of 58.3 minutes (Figure 6B). This delay in ES fusion kinetics was significant when compared with acute and CP clones (*P*<.0001 and *P*<.0001 respectively, One-way ANOVA). Therefore, even in the presence of saturating levels of CD4 and CCR5, ES clones do not complete entry processes as efficiently and exhibit slower kinetics than both CP and acute *env* clones.

This delay in entry kinetics was maintained during subsequent steps of the replication cycle as indicated by a kinetic reverse transcription assay. For these analyses, Efavirenz (a non-nucleoside reverse transcriptase inhibitor) was added at various times post-infection to arrest infection events which have not completed reverse transcription. As expected, ES derived *env* clones completed reverse transcription slower (mean T_1/2_ of 8.89 hours) than acute and CP clones [mean T_1/2_ values of 7.74 (*P*<.0001) and 7.95 (*P*<.0001) respectively with One-way ANOVA] (Figure 6C). Due to the isogenic background of the pseudotyping virus these results suggest that delays in reverse transcription are the result of delays in entry processes. ES *env* clones exhibit a kinetic lag in entry processes which are maintained during downstream events in the viral life cycle.

## Discussion

This study represents an evaluation of intrinsic phenotypic characteristics of full-length functional subtype B *env* quasispecies derived from ES plasma. Envelope glycoproteins from ES clearly exhibited reduced capacity to support HIV-1 entry into host cells compared to CP *env*s. Given the wide range in entry efficiencies observed with *envs* derived from acute infections it is possible that relatively lower fitness *env* variants are selected early in infection in ES. The impact of this observed entry deficiency with ES clones is still not fully understood but decreased replicative fitness and lack of diversification in ES viruses suggests these individuals may have contracted a less fit HIV-1 variant or these low fitness variants are selected for early in infection[7],[44],[45].

To date, phenotypic studies of ES viruses have been difficult to perform due to the low amount of virus in these individuals. Analysis of minor differences in *env* function has been confounded by the use of cell lines expressing non-physiologic amounts of co-receptor (CCR5). Given the high degree of variability in expression of CCR5 among patients it is important to evaluate *env* function over a wide range of CCR5 levels [26],[27],[34],[35]. Detailed analyses of *env* function in the presence of physiologic levels of CD4 and CCR5 was possible in this study through the use of the novel Affinofile system. Although ES and chronic individuals each harbored quasispecies with different receptor utilization phenotypes, ES clones from each individual showed an average decreased entry efficiency compared to chronic clones over almost all CD4 and CCR5 expression levels. These differences between chronic and ES clones were most dramatic at low CCR5 surface levels. Thus, this low fitness phenotype could be further accentuated *in vivo* in an individual expressing low CCR5 levels or possibly higher levels of CCR5 ligands which have been associated with viral control [46]–[48].

Several reports have suggested a correlation between susceptibility to entry inhibitors and relative *env* fitness [25],[40]. ES, CP, and acute clones exhibited a diverse range of IC_50_ values consistent with previous data [40],[41],[43],[49],[50]. However, acute clones showed consistently the most variation in susceptibility indicating the diverse phenotypes associated with early infection [40],[43],[49]. Conversely, the low range of IC_50_ values for ES clones underscores their phenotypic homogeneity. Due to reported differences between primary cells and cell lines entry inhibitor susceptibility assays were performed in the Affinofile cells induced to express CD4 levels and CCR5 levels that closely mimic primary CD4^+^ T cells (approximately 125,000 molecules CD4/cell and approximately 1274 molecules CCR5/cell) [51]. Additionally, it would be expected that variations in CD4 utilization would result in variations in susceptibility to soluble CD4 (sCD4) [52]. However, consistent with previous reports showing the relative resistance of primary isolate viruses to sCD4 [53], meaningful inhibition of ES, CP, and acute infection envelopes was not observed at maximal achievable concentrations of sCD4 (25 µg/ml).

Previous studies have also highlighted an association between receptor utilization profiles and susceptibility to neutralizing antibodies. It is possible that ES *envs* display altered susceptibility to broadly neutralizing antibodies given their observed entry phenotype. Previous studies suggest that antibody binding and neutralization have a kinetic component [54]. It may potentially be generalized that slow fusing viruses, independent of the mechanism, may be more susceptible to neutralizing antibodies that act with a kinetic dependence. However, it has been shown that ES individuals generate low titers of neutralizing antibodies against autologous virus and thus the role of neutralizing antibodies in maintenance of low level viremia in ES is nominal [23].

The host entry process is thought to be a rate limiting step in HIV-1 replication. It was thus important to determine if poor entry efficiency by ES clones leads to a reduced rate of entry kinetics. ES *env* clones were found to fuse on average over 1.5 times slower than chronic or acute clones in the presence of saturating levels of both CD4 and CCR5. As result of poor entry efficiency, this kinetic delay was maintained during subsequent steps of the retroviral lifecycle. Compounded effects of inefficient CD4 and CCR5 usage by ES clones likely contributes to kinetic delays in entry processes and thus overall decreased replicative fitness. The delayed entry kinetics may be even more important *in vivo* if there is a limited time frame over which entry can occur due to competing inhibitory processes such as the binding of neutralizing antibodies or the presence of CCR5 ligands.

Despite genotypic differences in both the virus and the ES host, viral quasispecies in different ES individuals are remarkably similar phenotypically. This implies that poor envelope function is a common feature in ES individuals. This result is in sharp contrast to data from acute infection *envs* where clones exhibited much phenotypic diversity. Potentially, HIV-1 infection in ES may be established by lower fitness *env(s)* which are present in a subset of acutely infected individuals. Alternatively, HIV-1 infection in ES may be established by phenotypically diverse *envs* and early pressure from the immune results in the outgrowth of lower fitness escape variants. At present, no data exists on the natural history of acute infection of ES. It remains unclear whether these individuals experience typical high level viremia that is subsequently reduced to an undetectable setpoint or control their viral load from the onset of infection. It would be of great interest to be able to address this significant gap in our understanding of viral dynamics in elite control of viremia. Lower *env* fitness is likely not sufficient to mediate absolute viral suppression. Viral control could be achieved in those individuals who are also able to mount a potent immune response and/or are genetically predisposed to better control HIV-1 viremia. In these individuals, viral replication and diversification of early infection viruses required to achieve efficient receptor utilization by *env* quasispecies may never be attained. Lower fitness of other viral factors may also be contributing to reduced replication and lower viral load. Full understanding of the *in vivo* impact of lower *env* fitness in ES will require further study however this data underscores the important contribution of viral factors in elite HIV-1 suppression.

## Supporting Information

Figure S1 ES and chronic *envs* show no difference in Env virion incorporation.
(7.47 MB EPS)

Figure S2 Affinofile cells allow for the analysis of both CD4 and CCR5.
(3.03 MB EPS)

Figure S3 Full surface plots for chronic progressor (CP) and elite suppressor (ES) envelopes. (A–O) Surface plots are arranged by patient, including Yu-2 and SF-162 control strains, indicated in blue. CP *envs* are indicated in grayscale, and ES *envs* are indicated in red.
(1.45 MB PDF)

Figure S4 Affinofile cells induced to express physiologic levels of CD4 and CCR5 express similar surface levels as CD4^+^ PBMCs.
(0.80 MB EPS)
