# Supplementary material for: Elite Suppressor–Derived HIV-1 Envelope Glycoproteins Exhibit Reduced Entry Efficiency and Kinetics
Source: PLoS Pathog. 2009 Apr 10;5(4):e1000377. doi: 10.1371/journal.ppat.1000377 (PMC2661022; doi:10.1371/journal.ppat.1000377)
Supplement: Figure S3 — Full surface plots for chronic progressor (CP) and elite suppressor (ES) envelopes. (A–O) Surface plots are arranged by patient, including Yu-2 and SF-162 control strains, indicated in blue. CP envs are indicated in grayscale, and ES envs are indicated in red. (1.45 MB PDF) [file ppat.1000377.s003.pdf]

Supplementary Figure 3

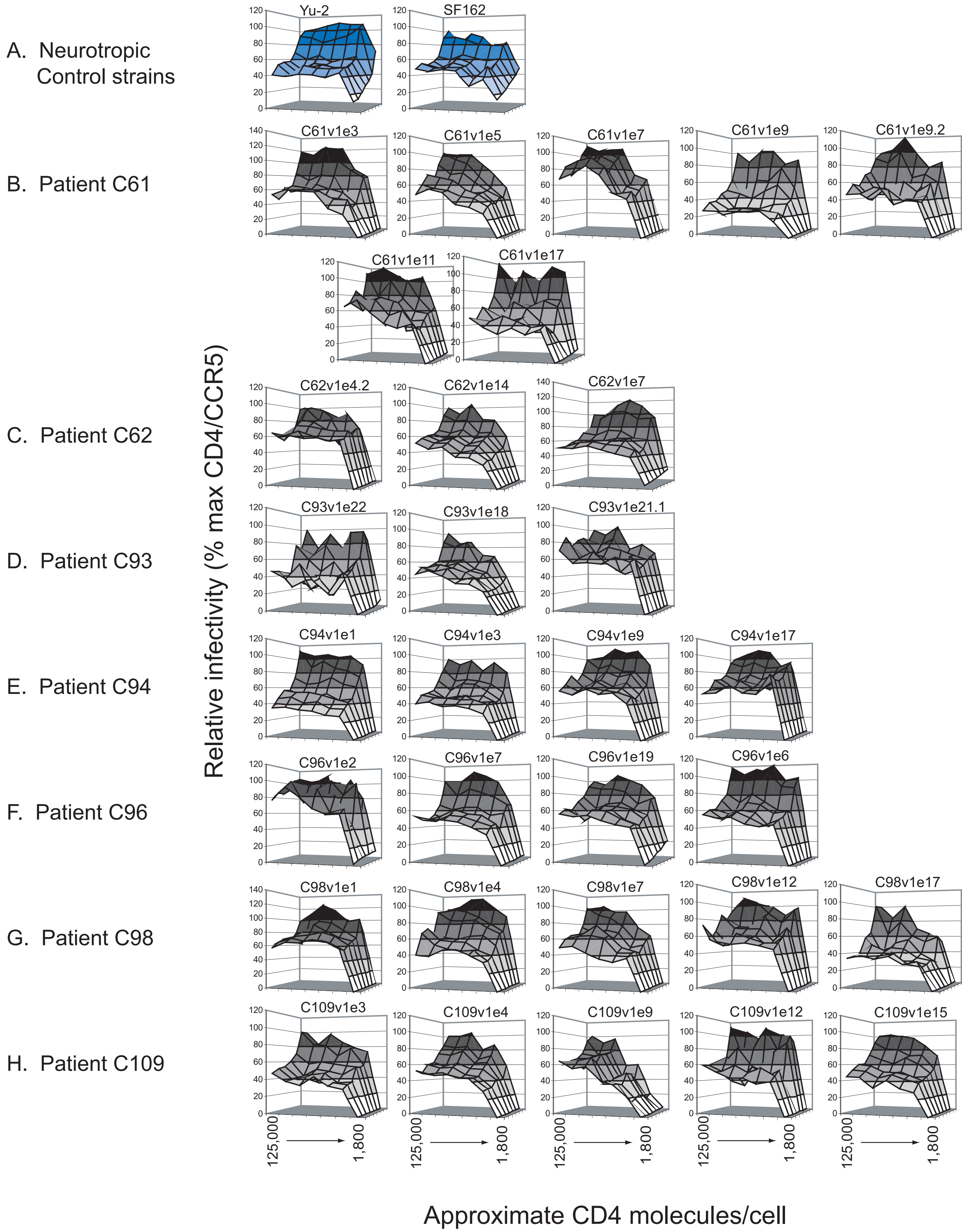

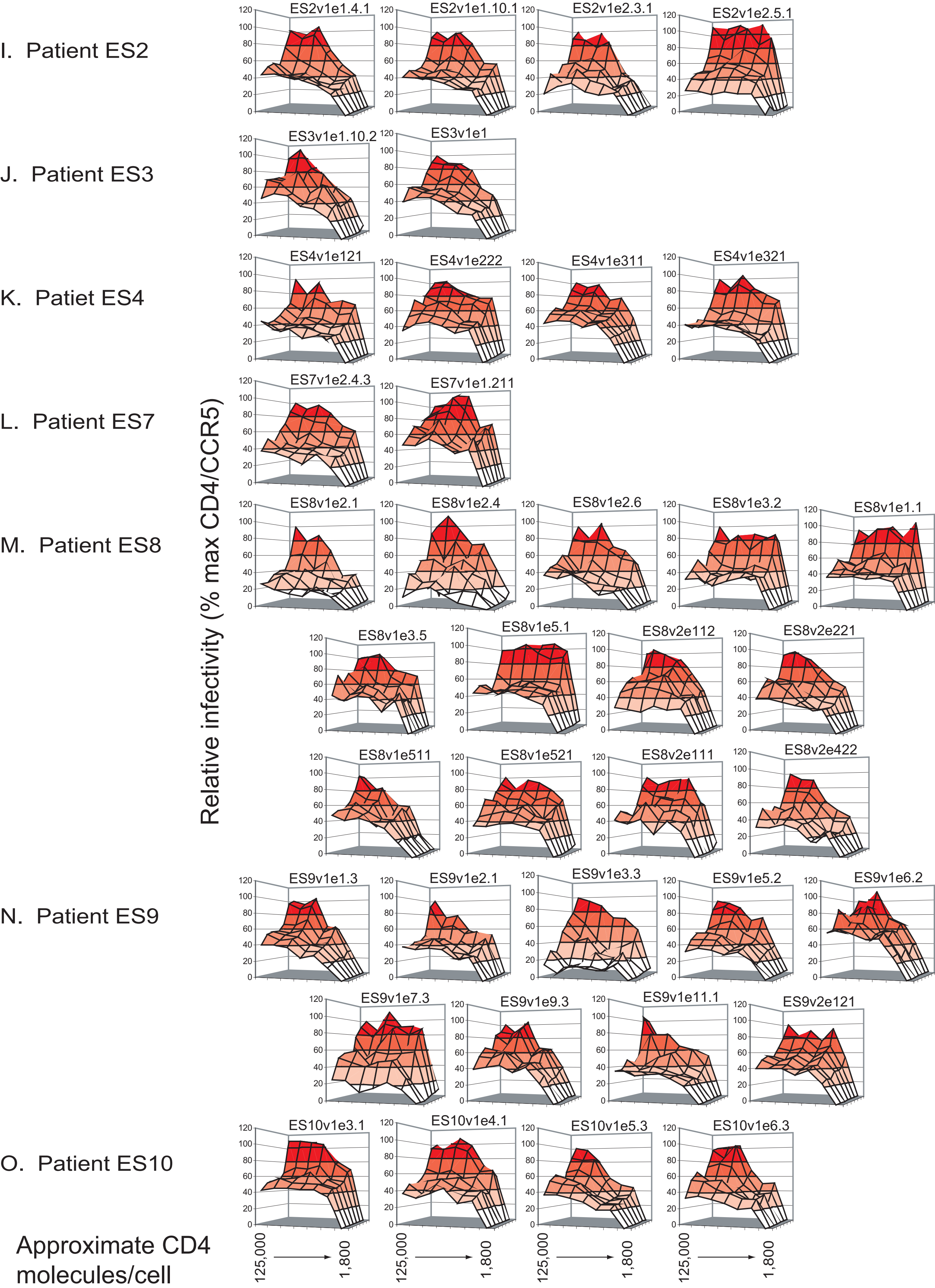

I. Patient ES2
ES2v1e1.4.1
ES2v1e1.10.1
ES2v1e2.3.1
ES2v1e2.5.1
J. Patient ES3
ES3v1e1.10.2
ES3v1e1
K. Patiet ES4
ES4v1e121
ES4v1e222
ES4v1e311
ES4v1e321
L. Patient ES7
ES7v1e2.4.3
ES7v1e1.211
M. Patient ES8
ES8v1e2.1
ES8v1e2.4
ES8v1e2.6
ES8v1e3.2
ES8v1e1.1
ES8v1e3.5
ES8v1e5.1
ES8v2e112
ES8v2e221
ES8v1e511
ES8v1e521
ES8v2e111
ES8v2e422
N. Patient ES9
ES9v1e1.3
ES9v1e2.1
ES9v1e3.3
ES9v1e5.2
ES9v1e6.2
ES9v1e7.3
ES9v1e9.3
ES9v1e11.1
ES9v2e121
O. Patient ES10
ES10v1e3.1
ES10v1e4.1
ES10v1e5.3
ES10v1e6.3
Relative infectivity (% max CD4/CCR5)
Approximate CD4 molecules/cell
125,000
1,800

Supplementary Figure 3 Full surface plots for chronic progressor (CP) and elite suppressor (ES) envelopes. (A-O) Surface plots are arranged by patient, including Yu-2 and SF-162 control strains, indicated in blue. CP envs are indicated in grayscale, and ES envs are indicated in red.
